# Long-term outcomes of PD-1 inhibitors plus chemotherapy as first-line treatment for advanced HER2-negative gastric cancer: an updated systematic review and meta-analysis

**DOI:** 10.3389/fimmu.2025.1651176

**Published:** 2025-11-18

**Authors:** Juping Tong, Shanmei Zhou, Shan Yin, Xiaojian Wang, Xinyi Liang, Jieru Quan, Duo Zhang, Shanguang Wu, Yilun Wei

**Affiliations:** 1 The First Affiliated Hospital of Guangxi University of Science and Technology, Guangxi University of Science and Technology, Liuzhou, Guangxi, China; 2 School of Economics and Management, Guangxi University of Science and Technology, Liuzhou, Guangxi, China; 3 Guangxi College Key Laboratory of Innovation Research on Medical and Engineering Integration, Guangxi University of Science and Technology, Liuzhou, Guangxi, China

**Keywords:** gastric cancer, chemotherapy, PD-1 inhibitors, overall survival, progression-free survival, objective response rate, treatment-related adverse events, meta-analysis

## Abstract

**Introduction:**

This meta-analysis was designed to compare the long-term outcomes of first-line programmed cell death protein 1 (PD-1) inhibitors plus chemotherapy versus chemotherapy in patients with advanced HER2-negative gastric cancer (GC).

**Materials and methods:**

Four databases (PubMed, Embase, Web of Science, and the Cochrane Library) were searched for randomized controlled trials (RCTs) comparing first-line PD-1 inhibitors plus chemotherapy to chemotherapy in patients with advanced HER2-negative GC. The search was conducted from the databases establishment to October 11, 2025. Overall survival (OS), progression-free survival (PFS), objective response rate (ORR), treatment-related adverse events (TRAEs), and Grade≥3 TRAEs were subjected to meta-analyses.

**Results:**

Six RCTs were included in the meta-analysis. The meta-analysis included a group of 6038 patients diagnosed with untreated advanced HER2-negative GC. Within this cohort, 3026 patients were administered first-line PD-1 inhibitors together with chemotherapy, while 3012 patients received first-line chemotherapy alone. Compared with chemotherapy, first-line PD-1 inhibitors plus chemotherapy yielded superior OS (HR = 0.91; 95% CI 0.89 to 0.93; P<0.01), PFS (HR = 0.89, 95%CI 0.87 to 0.91 P<0.01), and ORR (RR = 1.22, 95% CI, 1.16 to 1.29, P<0.01). With regards to safety, first-line PD-1 inhibitors plus chemotherapy exhibited a greater likelihood of encountering TRAEs (RR = 1.02, 95% CI: 1.00 to 1.04, P = 0.03) and Grade≥3 TRAEs (RR = 1.16, 95% Cl: 1.08 to 1.25, P<0.01) in comparison to chemotherapy.

**Conclusions:**

Compared with chemotherapy alone, PD-1 inhibitors plus chemotherapy as first-line therapy provided improved OS, PFS, and ORR. Furthermore, the heightened efficacy of PD-1 inhibitors plus chemotherapy was accompanied by a rise in TRAEs and Grade≥3 TRAEs.

**Systematic review registration:**

https://www.crd.york.ac.uk/prospero/, identifier CRD420251015248.

## 1 Introduction

Gastric cancer (GC) is the fifth leading cause of cancer-related deaths worldwide (1). In 2022, there were over 968,000 new cases of advanced GC and close to 660,000 deaths, ranking the disease as fifth in terms of both incidence and mortality worldwide (1). Among men, it is the most frequent cancer and the leading cause of cancer death in several South-Central Asian countries, including Afghanistan, Iran, Kyrgyzstan, and Tajikistan (1). GC is rampant in many parts of the world and is often diagnosed at advanced stages (2). Despite the high incidence of GC, most patients are unfortunately diagnosed at advanced stages with dismal prognoses due to the lack of distinctive clinical indications (3, 4). GC can be classified into four molecular subtypes, one of which is chromosomal instability (CIN) (5). This subtype is associated with HER2 protein (5). Overexpression of the ERBB2(human epidermal growth factor receptor 2, formerly known as HER2) protein has been implicated in the development of gastric adenocarcinoma (6).

Fluoropyrimidine- and platinum-based chemotherapy remains the first-line standard of care for HER2-negative, advanced GC and this treatment has not changed substantially over the past decade (2). The recommended platinum compounds include oxaliplatin or cisplatin, with oxaliplatin being the preferred option, particularly for older patients. Recommended fluoropyrimidines are intravenous 5-FU, oral capecitabine, or oral S-1. Fluoropyrimidine monotherapy or a fluoropyrimidine in combination with irinotecan or a taxane can be considered as an alternative option for patients who do not tolerate platinum compounds (7). The median overall survival (OS) for advanced-stage GC treated with conventional chemotherapy is less than one year (8).

Immunotherapy is considered an effective therapeutic strategy in medical oncology. Immune checkpoint inhibitors (ICIs), which target pathways involved in immune regulation, disrupt the cycle of immune tolerance and allow T-cell recognition against tumor cells, thereby increasing the immune response of immune cells to cancer and inhibiting the immune evasion induced by cancer cells (9, 10). Immune checkpoint inhibitors (ICPIs), such as PD-1 are new cancer drugs. The ATTRACTION-2 trial, the first randomized, double-blind, placebo-controlled, phase III study, demonstrated the efficacy and safety of nivolumab, a humanized anti-PD-1 monoclonal antibody, as a third- or later-line treatment for patients with advanced gastric cancer (AGC) (11). Nivolumab provided an improvement in OS compared with placebo (median OS: 5.26 vs 4.14 months; hazard ratio (HR)= 0.63, P<0.01) (11). The KEYNOTE-059 study was an open-label, nonrandomized, 3-cohort, phase II trial in which 259 patients were enrolled in cohort 1 to investigate pembrolizumab monotherapy as a third- or later-line treatment for patients with AGC (12). Pembrolizumab showed similar efficacy to nivolumab (objective response rate (ORR), 11.6%; median OS, 5.60 months (95% CI, 4.3 to 6.9); median PFS, 2.00 months (95% CI, 2.0 to 2.1) (12). PD‐1 monoclonal antibodies were approved for third‐line treatment of advanced GC (13). However, the efficacy of immune checkpoint inhibitors alone is limited (13). Nivolumab plus fluoropyrimidine- and oxaliplatin-based chemotherapy is a preferred first-line treatment option for patients with HER2 negative gastric tumors with programmed cell death ligand 1(PD-L1) expression levels by combined positive score(CPS) of ≥5 (category 1) and is useful under certain circumstances for tumors with PD-L1 expression levels by CPS of <5 (2). For patients with HER2-postive disease, the recommended first-line regimen is trastuzumab (anti-HER2) in combination with platinum and fluoropyrimidine-based chemotherapy (2). Thus, PD-1 inhibitors plus chemotherapy for advanced HER2-negative GC demonstrates efficacy. The combination of PD‐1 monoclonal antibody and chemotherapy has now become the new standard for first‐line treatment of advanced metastatic GC in China (13). Chemotherapy induces immunogenic cell death (ICD), releasing tumor antigens and danger signaling molecules that reconfigure the tumor immune microenvironment (TIME) (14). This process leads to significant increases in CD3+ T cells, CD8+ T cells, and natural killer (NK) cells, while reducing regulatory T cells (Tregs) (15). Within this reconfigured microenvironment, PD-1 inhibitors alleviate T cell exhaustion and enhance immune response (15). PD-1 inhibitors and chemotherapy have synergistic effects to enhance the therapeutic effect (14).

However, there was still no meta-analysis evaluating the long-term outcomes of PD-1 inhibitors combined with chemotherapy as first-line treatment for advanced HER2-negative GC. The highest level of evidence-based medicine (high-quality meta-analysis) can further confirm the benefit-to-risk ratio, provide a more solid “ironclad” for guideline recommendations, and may influence the recommendation level. Therefore, this meta-analysis was designed to evaluate the long-term outcomes of PD-1 inhibitors combined with chemotherapy as first-line treatment for advanced HER2-negative GC.

## 2 materials and methods

### 2.1 Search strategy

In compliance with the 2020 guidelines of the Preferred Reporting Project for Systematic Review and Meta-Analysis (PRISMA), the current meta-analysis was conducted (16). This study was registered with PROSPERO under the number CRD420251015248. A systematic search was conducted in four databases—PubMed, Embase, Web of Science, and the Cochrane Library, to identify literature published up to October 11, 2025. The search strategy utilized a combination of medical subject headings (MeSH) and free-text words following the PICOS principle. The search keyword included “gastric cancer” AND “PD-1 inhibitors” AND “randomized controlled trial” AND “metastatic”. **Supplementary Material 1** provided a comprehensive listing of the search results.

### 2.2 Inclusion and exclusion criteria

The criteria for inclusion were as follows (1): patients with untreated advanced HER2-negative GC; (2) patients in the intervention group received PD-1 inhibitors in combination with chemotherapy as first-line treatment; (3) patients in the controlled group received chemotherapy as first-line treatment; (4) at least one of the following outcomes were reported: OS, progression-free survival (PFS), ORR, treatment-related adverse events (TRAEs), and grade 3 or higher (Grade≥3) TRAEs; (5) study types: randomized controlled trials (RCTs); (6) Median follow-up time was at least 36 months.

The criteria for exclusion were as follows: (1) other types of articles, such as case reports, publications, letters, comments, reviews, meta-analyses, editorials, animal studies, protocols, and conference; (2) other diseases; (3) not relevant; (4) Reduplicate cohort of patients; (5) failed to extract data.

### 2.3 Selection of studies

The selection of studies, including elimination of duplicates, was undertaken using EndNote (Version 20; Clarivate Analytics). An initial screening was performed by two reviewers who independently removed duplicate entries, evaluated the titles and abstracts for relevance, and classified each study as either included or excluded. Discrepancies were resolved through consensus. A third reviewer of the review would take on the role of an arbitrator if lacking a consensus.

### 2.4 Data extraction

Two independent reviewers conducted a meticulous analysis of the title and abstract, followed by a detailed review of the full texts. A third investigator was consulted to resolve inconsistencies. The data collected included the name of the trial identifier, author (year), study design, countries, groups, regimens, number of patients, median age, gender, microsatellite instability-high (MSI-H), eastern cooperative oncology group performance status (ECOG PS), PD-L1 expression, OS, PFS, ORR, TRAEs, Grade≥3 TRAEs.

### 2.5 Risk of bias assessment

Two independent reviewers independently evaluated the methodological quality of each individual study, using the Cochrane risk of bias tool (RoB 1.0 Tool) (17), which included seven domains (1): random sequence generation; (2) allocation concealment; (3) blinding of participants and personnel; (4) blinding of outcome assessment; (5) incomplete outcome data; (6) selective reporting; (7) others bias. The quality assessment results determined the labeling of each feature as low, unclear, or high risk. The quality assessment was conducted by two independent reviewers, and any discrepancies were resolved through consultation with a third reviewer.

### 2.6 Statistical analysis

The removal of duplicate studies was conducted using EndNote (Version 20; Clarivate Analytics). Statistical analysis was performed using the Review Manager v5.3 software. The fixed-effects model incorporates the odds ratio (OR) for binary outcomes and the mean difference (MD) for continuous outcomes in its initial structure. These metrics are reported together with their corresponding 95% confidence intervals (CI). For continuous data, medians and interquartile ranges were transformed into means and standard deviations. Although the chi-square test (P < 0.1) or the I² test (with results larger than 50%) indicate substantial heterogeneity, we use the random-effects model to clarify the variability. Statistical significance was defined as a p-value less than 0.05.

## 3 Results

### 3.1 Search results

**Figure 1:**
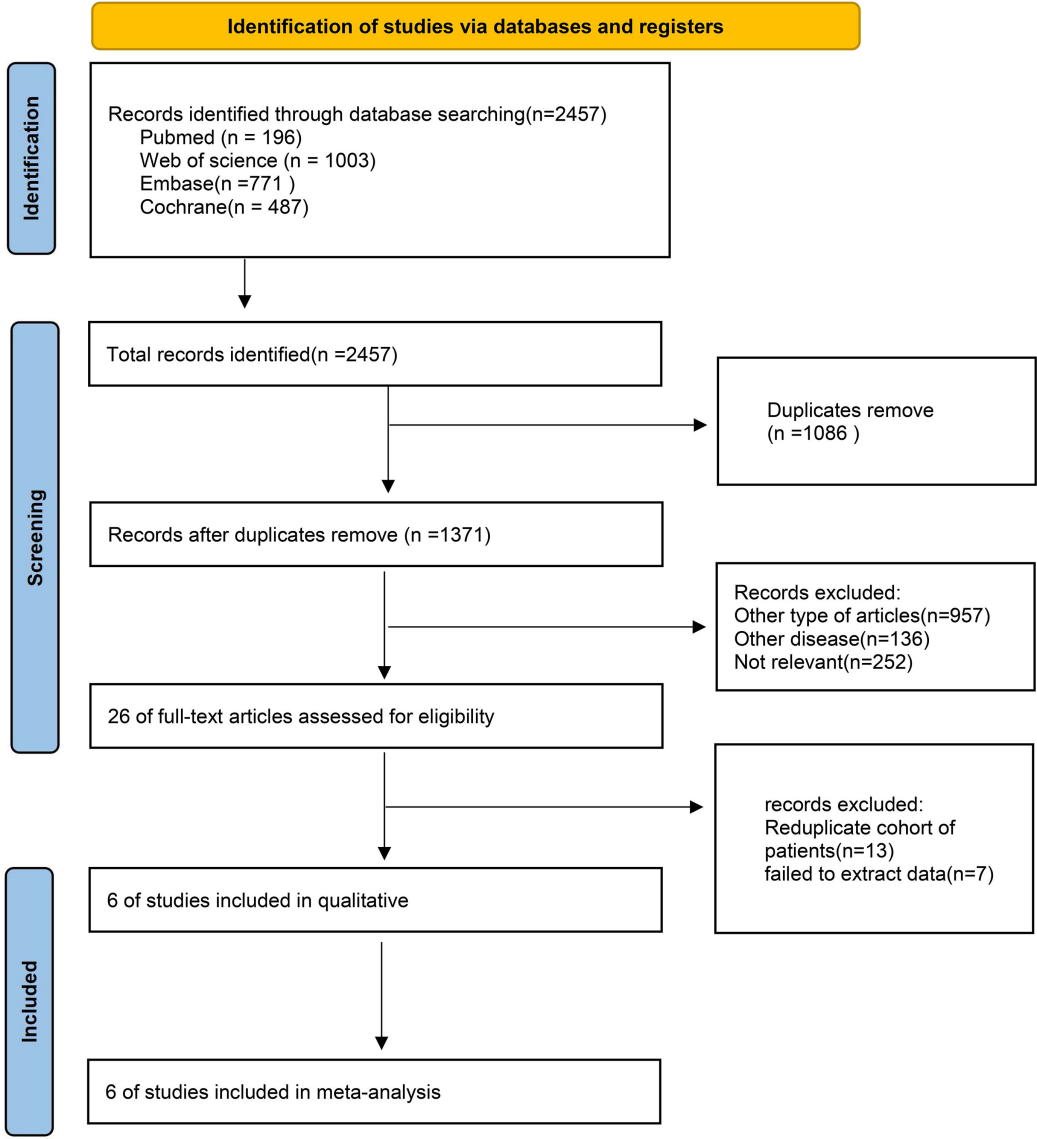
Flow chart of literature search strategies.

The process of literature selection and inclusion is illustrated in **Figure 1**. Initially, 2000 records were identified. Following the removal of superfluous research, a grand total of 1184 papers remained. Based on the evaluation of the titles and abstracts, a total of 1178 publications were considered unsuitable and so eliminated. A full-text review was conducted, resulting in the inclusion of six RCTs in this meta-analysis.

### 3.2 Patient characteristics

This meta-analysis included six RCTs (18–23) published between 2020 and 2025. These trials enrolled a total of 6038 patients diagnosed with untreated advanced HER2-negative GC. Among these patients, 3026 patients were administered first-line PD-1 inhibitors plus chemotherapy, while 3012 patients received first-line chemotherapy alone. The Trial identifier, author(year), countries, groups, regimens, number of patients, median age, gender, MSI-H, ECOG PS, and PD-L1 expression were shown in **Table 1**.

**Table 1:** Basic characteristics of included trials.

Trial identifier	Author (year)	Countries	Median follow-up period (month)	Groups	Regimens	Number of patients	Median age (year)	Gender (female ,%)	MSI-H (%)	ECOG PS	PD-L1 expression
										1 (%)	positive (%)
ATTRACTION-4 (18)	Narikazu Boku (2024)	Japan, South Korea, China	40	A	Nivolumab, 360mg, Q3W	362	64 (25-86)	253 (70.0)	NR	167 (46)	58 (16)
				B	SOX or CAPOX	362	65 (27-89)	270 (75.0)	NR	168 (46)	56 (15)
CheckMate 649 (19)	Yelena Y. Janjigian (2025)	Multinational (11 countries)	60	A	Nivolumab, 360mg, Q3W or 240mg, Q2W	789	62 (18-88)	540 (68.0)	23 (3)	461 (58)	126 (16)
				B	XELOX or FOLFOX	792	61 (21-90)	560 (71.0)	21 (3)	452 (57)	127 (16)
KEYNOTE-062 (20)	Kohei Shitara (2022)	Multinational (29 countries)	54	A	Pembrolizumab, 200 mg, Q3W	257	62 (22-83)	195 (75.9)	17 (6.6)	138 (53.7)	257 (100)
				B	SOC	250	62.5 (23-87)	179 (71.6)	19 (7.6)	135 (54)	250 (100)
KEYNOTE-859 (21)	Sun Young Rha (2025)	Multinational (33 countries)	55	A	Pembrolizumab, 200mg, Q3W	790	61 (52-67)	527 (67.0)	39 (5)	509 (64)	618 (78)
				B	FP or CAPOX	789	62 (52-69)	544 (69.0)	35 (4)	488 (62)	617 (78)
ORIENT-16 (22)	Jianming Xu (2023)	China	36	A	Sintilimab, <60 kg 3 mg/kg or ≥60kg 200mg/kg, Q3W	327	62 (55-67)	253 (77.4)	NR	238 (72.8)	197 (60.2
				B	XELOX	323	60 (52-67)	230 (71.2)	NR	232 (71.8)	200 (61.9)
RATIONALE-302 (23)	Miao-Zhen Qiu (2024)	Multinational (12 countries)	36	A	Tislelizumab,200mg,Q3W	501	60.0 (53-66)	346 (69.0)	16 (3)	332 (66)	274 (55)
				B	Oxaliplatin + capecitabine or cisplatin + 5-Fluorouracil	496	61.0 (54-68)	346 (70.0)	24 (5)	342 (69)	272 (55)

A, PD-1inhibitors plus chemotherapy group; B, chemotherapy group; Q3W, every 3 weeks; Q2W, every 2 weeks; SOX, oxaliplatin+tegafur–gimeracil–oteracil potassium; CAPOX, oxaliplatin+capecitabine; XELOX, capecitabine+oxaliplatin; FOLFOX; leucovorin+fluorouracil+oxaliplatin; SOC, cisplatin+fluorouracil or capecitabine; FP, fluorouracil+cisplatin; CAPOX, capecitabine+oxaliplatin; NR, Not reported; MSI-H, microsatellite instability-high; ECOG PS, eastern cooperative oncology group performance status.

### 3.3 Risk of bias

**Figure 2:**
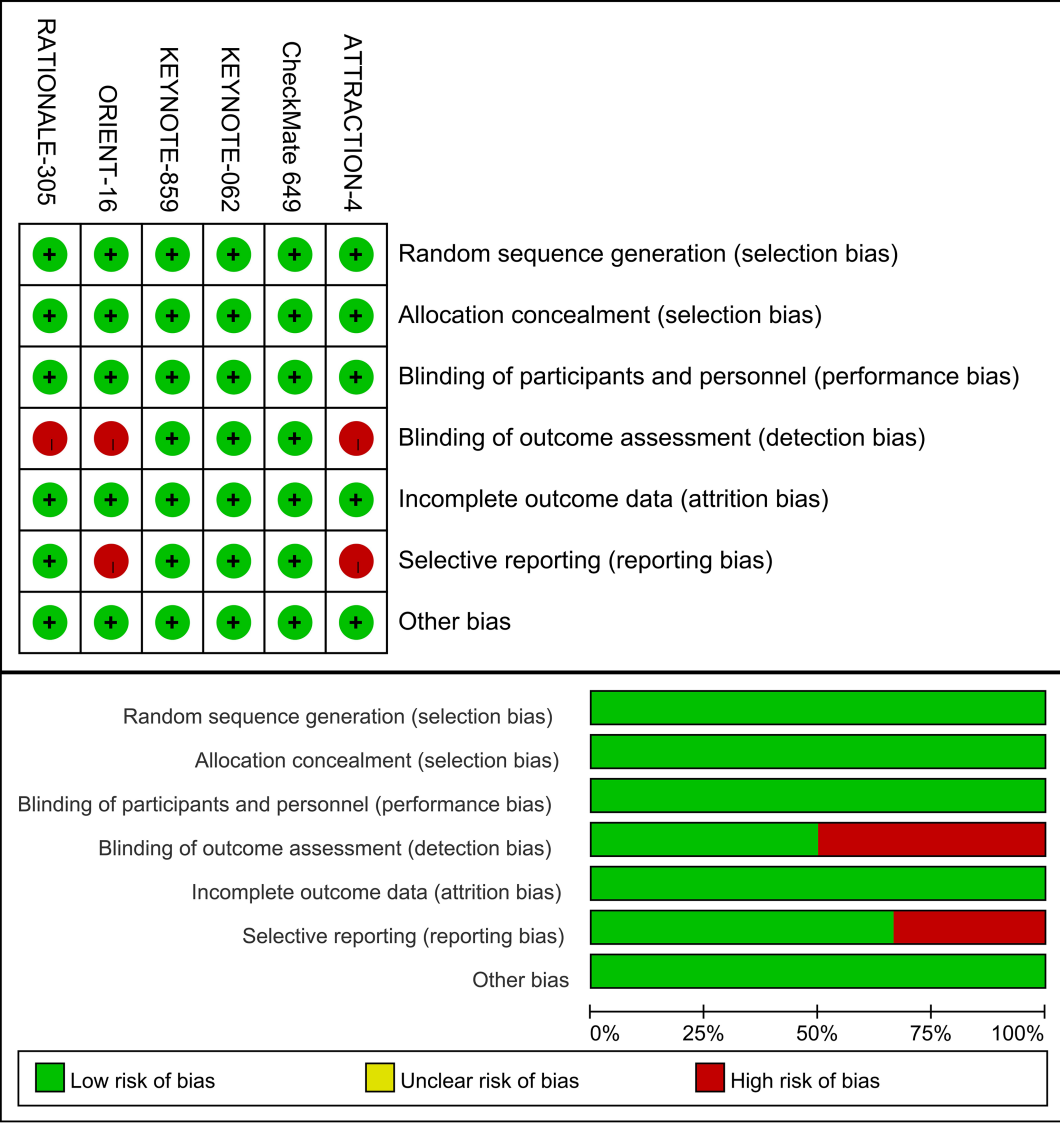
Risk of bias assessment diagram.

**Figure 2** provides a summary of the risk of bias assessment results. Six studies produced a sufficient random sequence, reported appropriate allocation concealment, clearly implemented participant blinding, provided complete outcome data, and did not exhibit any other bias. Three studies reported outcome assessor blinding and four studies did not engage in selective reporting.

### 3.4 Efficacy outcomes

#### 3.4.1 OS

**Figure 3:**
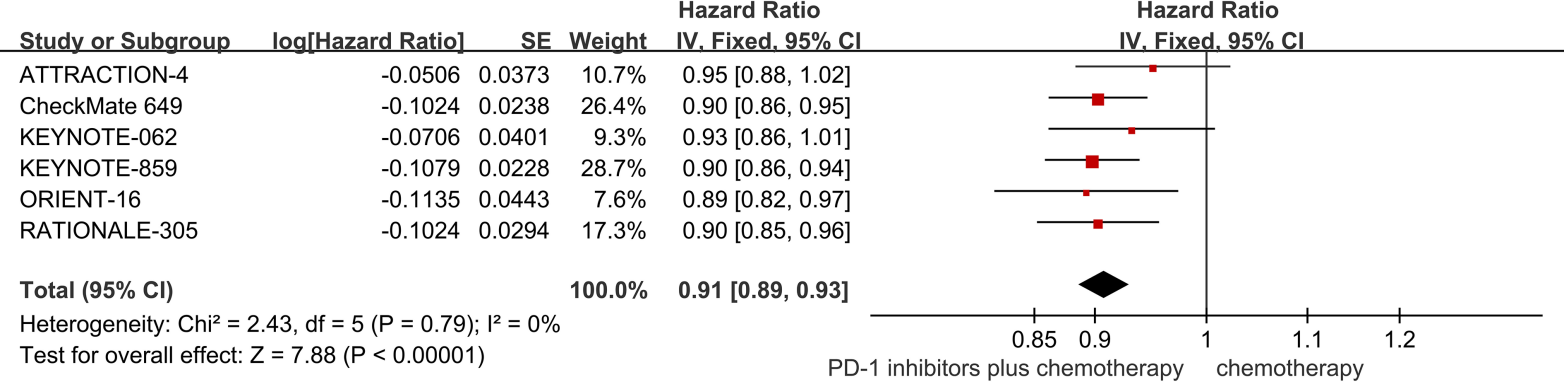
Forest plot of the meta-analysis for OS.

Six studies provided data regarding OS (18–23). The pooled result showed that PD-1 inhibitors plus chemotherapy significantly improved OS compared with chemotherapy (HR = 0.91; 95% CI 0.89 to 0.93; P<0.01) (**Figure 3**).

Subgroup analyses were conducted regarding PD-L1 expression, age, and gender (**Table 2**). It was worth noting that, PD-1 inhibitors plus chemotherapy significantly improved OS compared with chemotherapy regardless of the PD-L1 expression, age, and gender of the patients.

**Table 2:** Results of subgroup analyses for OS.

Groups	No.of studies	HR (95%CI)	P	I2(%)
PD-1 inhibitors plus chemotherapy VS chemotherapy				
Total	6	0.91(0.89,0.93)	0.81	0
Age(yrs)				
<65 years old	6	0.90(0.87,0.93)	0.96	0
≥65 years old	6	0.91(0.88,0.95)	0.47	0
Gender				
Male	6	0.90(0.87,0.93)	0.9	0
Female	6	0.92(0.88,0.96)	0.74	0
PD-L1 expression				
Negative	5	0.94(0.91,0.97)	0.9	0
Positive	6	0.87(0.84,0.90)	0.06	53

#### 3.4.2 PFS

**Figure 4:**
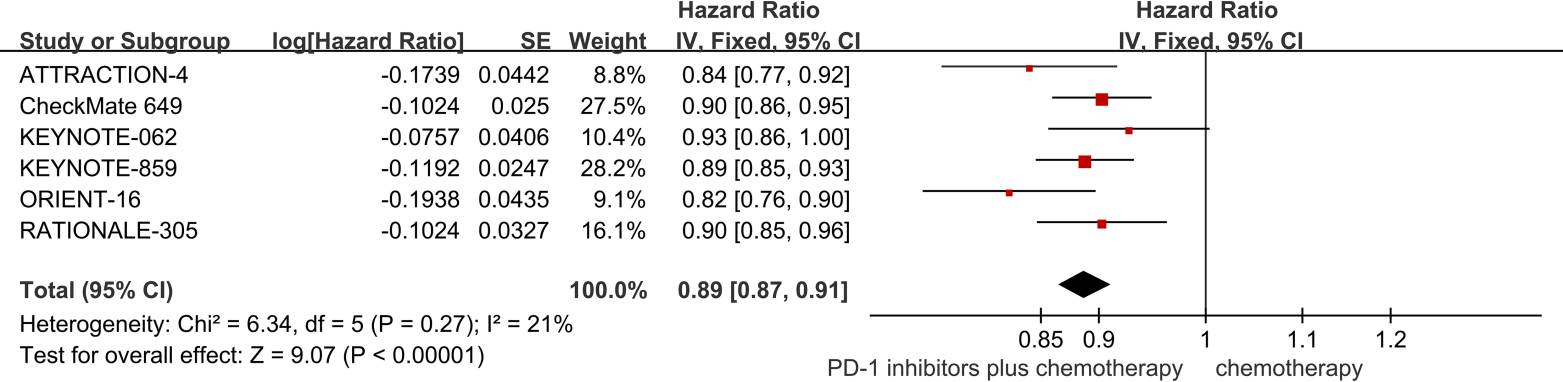
Forest plot of the meta-analysis for PFS.

The six RCTs included in the analysis provided data on PFS (18–23). PD-1 inhibitors plus chemotherapy resulted in improved PFS compared to chemotherapy (HR = 0.89, 95% CI 0.87 to 0.91 P<0.01) (**Figure 4**).

#### 3.4.3 ORR

**Figure 5:**
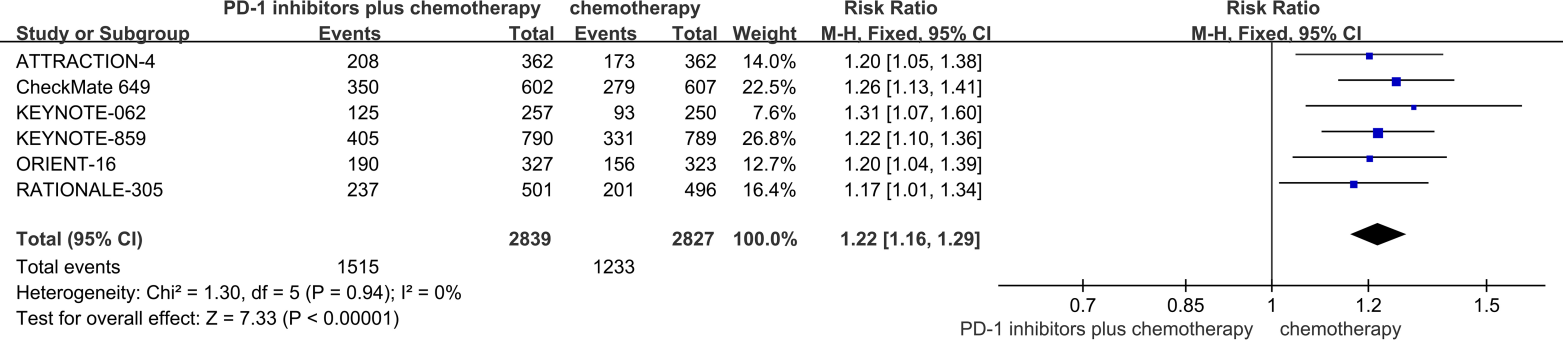
Forest plot of the meta-analysis for ORR.

Among the six RCTs considered, six studies provided data regarding ORR (18–23). The ORR of patients in PD-1 inhibitors plus chemotherapy group was significantly higher than that of the chemotherapy group (53.4% vs 43.6%, risk ratio (RR) = 1.22, 95% CI, 1.16 to 1.29, P<0.01) (**Figure 5**).

### 3.5 Safety outcomes

**Figure 6:**
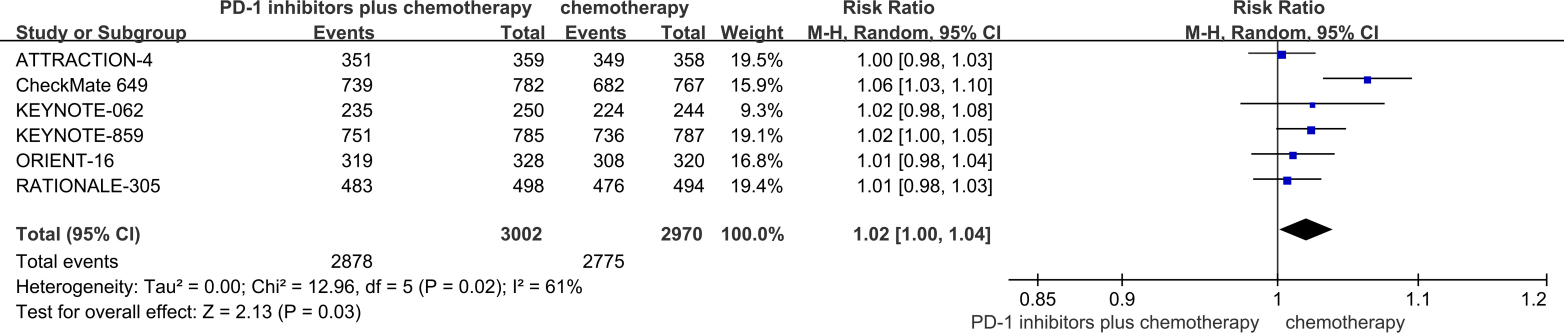
Forest plot of the meta-analysis for TRAEs.

Out of the six RCTs examined, six trials presented data on TRAEs (18–23). With regards to safety, first-line PD-1 inhibitors plus chemotherapy exhibited a greater likelihood of encountering TRAEs (95.9% vs 93.4%, RR = 1.02, 95% CI: 1.00 to 1.04, P = 0.03) (**Figure 6**) in comparison to chemotherapy. The TRAEs with the highest incidence were nausea, anemia, neutropenia, thrombocytopenia, and decreased appetite.

**Figure 7:**
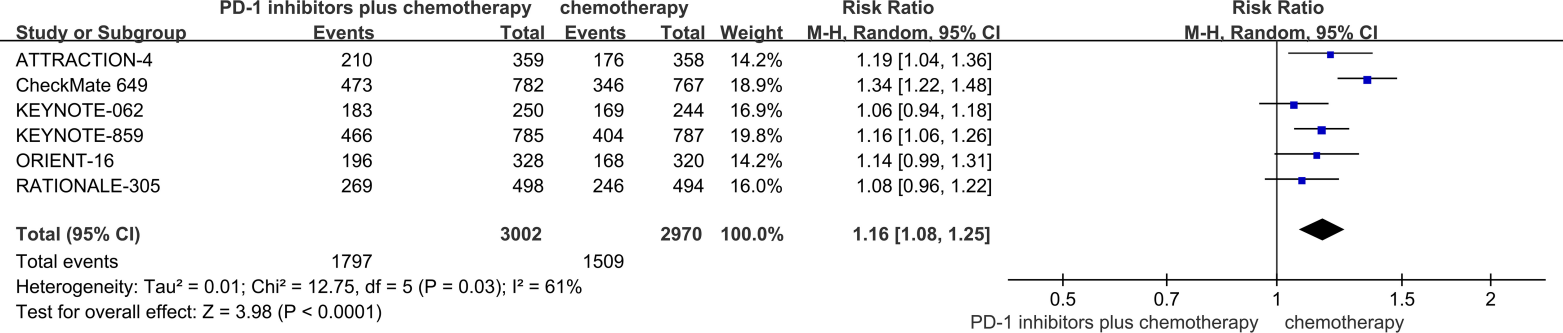
Forest plot of the meta-analysis for Grade≥3 TRAEs.

Based on the analysis of six RCTs (18–23), the incidence of Grade≥3 TRAEs was significantly higher in patients receiving PD-1 inhibitors combined with chemotherapy compared to those receiving chemotherapy alone (59.9% vs 50.8%, RR = 1.16, 95% Cl: 1.08 to 1.25, P<0.01) (**Figure 7**). The Grade≥3 TRAEs with the highest incidence were thrombocytopenia, decreased neutrophil count, anemia.

### 3.6 Publication bias

An evaluation of publication bias was conducted using four funnel plots. The bilateral symmetric funnel plots of the OS (**Supplementary Figure S1**), PFS (**Supplementary Figure S2**), ORR (**Supplementary Figure S3**), TRAEs (**Supplementary Figure S4**), and Grade≥3 TRAEs (**Supplementary Figure S5**) did not reveal any significant evidence of publication bias.

## 4 Discussion

### 4.1 General interpretation of the results in the context of other evidence

Over the past half-century, GC rates have exhibited a steady decline across most populations—a trend largely attributed to a reduction in non-cardia GC. This unanticipated success in prevention is primarily linked to advancements in food preservation and storage techniques, as well as a decline in the prevalence of *Helicobacter pylori* (*H. pylori*) infection (24). Although overall GC incidence rates are projected to continue decreasing, with the disease becoming increasingly uncommon in many countries, significant geographical disparities in incidence rates remain. Certain regions are anticipated to sustain elevated GC rates in the foreseeable future (25). The advent of PD-1 inhibitors has represented a significant breakthrough in the treatment of advanced GC, demonstrating notable anti-tumor efficacy in patients (26). While prior meta-analyses (27–30) had shown the effectiveness and safety of PD-1 inhibitors combined with chemotherapy as first-line treatment for advanced HER2-negative GC, they failed to include the updated information on the long-term outcomes of RCTs. Over the course of the last one year, multiple randomized controlled trials (RCTs) have updated their long-term outcomes (18, 19, 21, 23). Hence, it is both possible and essential to perform an updated meta-analysis that compares the long-term outcomes of PD-1 inhibitors combined with chemotherapy as first-line treatment for advanced HER2-negative GC. The majority of randomized controlled trials (RCTs) comprised in this updated meta-analysis assessed long-term outcomes over a follow-up period of at least three years. Therefore, compared with prior meta-analyses, this meta-analysis provided the updated information regarding long-term efficacy and safety of first-line PD-1 inhibitors plus chemotherapy in patients with advanced HER2-negative GC. The findings demonstrated that the PD-1 inhibitors plus chemotherapy significantly improved OS, PFS, and ORR.

Chemotherapy inhibits tumor growth primarily by arresting the cell cycle, inhibiting DNA replication, disrupting cell metabolism, and suppressing microtubule assembly (31). Certain cytotoxic chemotherapeutic agents, such as oxaliplatin, can induce immunogenic cell death and stimulate antitumor immune responses (32). Chemotherapy modifies the TIME (33). Tumor-specific cytotoxic T cells recognize cancer neoantigens, leading to tumor cell death through direct cytotoxicity and the release of inflammatory mediators. During this process, activated T cells upregulate the expression of PD-1 on their surface (34). PD-1 is a monomeric type I transmembrane immune checkpoint receptor, predominantly expressed on T cells, B cells, NK cells, and tumor-infiltrating lymphocytes (35). The binding of PD-1 to its ligand, programmed cell death ligand 1 (PD-L1), triggers an inhibitory signaling pathway, resulting in reduced T-cell proliferation and impaired antitumor immunity. PD-1 inhibitors block the interaction between PD-1 and its ligands (PD-L1 or PD-L2) by binding to overlapping surface regions of PD-1 (36). The efficacy of PD-1 inhibitors largely depends on the patient’s pre-existing immune response, which is influenced by the TIME (37). The combination of PD-1 inhibitors and chemotherapy exerts a synergistic antitumor effect (38). Preclinical studies in mouse models have shown that PD-1 inhibitors plus chemotherapy suppress tumor growth by enhancing the infiltration of antitumor immune cell subsets (39). In clinical trials, pembrolizumab demonstrated significant antitumor activity and was well tolerated both as monotherapy and in combination with chemotherapy in patients with previously untreated advanced gastric or gastroesophageal junction adenocarcinoma. The ORR was 60.0% (95% CI, 38.7 to 78.9) for combination therapy and 25.8% (95% CI, 11.9 to 44.6) for monotherapy (40). PD-1 inhibitors plus chemotherapy exhibit superior efficacy compared to chemotherapy alone.

Regarding safety, the incidence of TRAEs was higher in the PD-1 inhibitors plus chemotherapy group compared to the chemotherapy group. Similarly, the incidence of Grade ≥3 TRAEs was higher in the PD-1 inhibitors plus chemotherapy group compared to the chemotherapy group. The combination of PD-1 inhibitors with chemotherapy may lead to an increased occurrence of adverse events. The frequently observed TRAEs included nausea, anemia, neutropenia, thrombocytopenia, and decreased appetite, with nausea reported as the predominant event. Immune-related TRAEs, particularly those affecting the endocrine, gastrointestinal, and skin systems, were frequently observed during treatment (18–23). The Grade ≥3 TRAEs with the highest incidence were thrombocytopenia, decreased neutrophil count, and anemia. When common adverse reactions occur, symptomatic supportive treatment should be given. The prognosis was usually good after proper treatment. When severe or life-threatening TRAEs occur, treatment interruption or discontinuation was allowed, and alternative anti-cancer therapy should be administered. It is crucial to closely monitor patients’ vital signs during the administration of PD-1 inhibitors plus chemotherapy, remain vigilant for severe TRAEs, and promptly adjust treatment plans based on patients’ clinical conditions.

### 4.2 Limitations of the evidence included in the review

However, this study has several limitations. As with any meta-analysis, inherent variability across the included studies—such as differences in patient baseline characteristics, disease stage, and treatment protocols—may have influenced the results. This analysis included only six RCTs, highlighting the need for additional large-scale, well-designed RCTs to validate these findings. Furthermore, we were unable to evaluate other relevant outcomes, such as quality of life and indicators of carcinogenesis. Subgroup analyses were limited due to incomplete reporting and insufficient statistical power. For instance, OS data were missing for certain subgroups, such as non-Asian populations and MSI-H status. Similarly, PFS data for subgroups based on Asian versus non-Asian populations, PD-L1 expression levels, gender, and age were incompletely reported. Tumor responses were not assessed by a blinded independent central review committee, which may have introduced bias in outcomes ascertainment (22, 23). Discrepancies were observed between investigator-assessed PFS and centrally assessed PFS in some studies (18). Additionally, potential clinical heterogeneity exist across trials, such as geographic locations, chemotherapy regimens and PD-L1 scoring methods which might reduce the effectiveness and applicability of the results. Four included RCTs were conducted in multinational center, while another two RCTs were conducted only in Asian countries. The chemotherapy regimens were different among these RCTs, such as SOX, CAPOX, XELOX, SOC or XELOX. Regarding PD-L1 scoring methods, it could be tumor proportion score or combined positive score in different RCTs.

### 4.3 Limitations of the review processes used

Although a comprehensive systematic search was conducted across multiple databases, including PubMed, Embase, Web of Science, and the Cochrane Library, some studies may not have been identified due to potential limitations in the search strategy, such as variations in keyword usage. During the literature screening process, subjective judgments may have led to the omission of eligible studies. Additionally, unpublished negative results may not have been adequately captured. PFS data for certain subgroups, such as Asian versus non-Asian populations, PD-L1 expression levels, gender, and age, were incompletely reported. Notably, the ATTRACTION-4 and ORIENT-16 trials did not provide data on MSI-H status (18, 22). Despite attempts to contact the corresponding authors for additional information, no responses were received. This lack of data may introduce potential biases and limit the generalizability of findings.

### 4.4 Implications of the results for practice, policy, and future research

The findings demonstrated a significant benefit from the addition of PD-1 inhibitors to chemotherapy as first-line treatment in this patient population. Therefore, PD-1 Inhibitors plus chemotherapy should be recommended as first-line treatment for advanced HER2-negative gastric cancer. However, careful attention must be paid to the occurrence of TRAEs to ensure a balanced and reasonable treatment approach. Notably, a higher incidence of severe TRAEs was observed in the PD-1 inhibitors plus chemotherapy group. Therefore, it is crucial to closely monitor patients’ vital signs during the administration of PD-1 inhibitors plus chemotherapy, remain vigilant for severe TRAEs, and promptly adjust treatment plans based on patients’ clinical conditions. Consequently, the implementation of PD-1 inhibitors plus chemotherapy necessitates the proactive development of prevention and management strategies to mitigate potential severe TRAEs. The comprehensive outcomes and long-term prognostic data require further validation through subsequent studies. Additional exploration is needed to evaluate the optimal sequencing and selection of chemotherapy combinations in the first-line setting, as well as to determine the impact of first-line combination regimens incorporating PD-1 inhibitors on treatment response and survival outcomes.

Future studies should aim to provide more comprehensive subgroup analyses to better understand the differential treatment effects across diverse patient populations and characteristics. The assessment of outcome measures should be conducted through a centralized review process. Tumor response was assessed per response evaluation criteria in solid tumors (RECIST) version 1.1 by blinded independent central review (BICR). Disease progression was confirmed through central review. Investigators, participants, site staff, and funder personnel were masked to group assignment. Additional RCTs with robust trial designs and follow-up periods exceeding five years are needed to validate these findings. Moving forward, it is hoped that personalized precision immunotherapy, guided by population screening and treatment optimization, will provide greater clinical benefits to patients. The combination of PD-1 inhibitors plus chemotherapy has demonstrated remarkable efficacy and identifying optimal regimens, dosing strategies, and treatment protocols remains a major challenge. More precise biomarkers such as tumor mutational burden (TMB), genomic expression profile (GEP), and TIME should be investigated in the future. Systematic study and development of these biomarkers can better identify patients eligible for immunotherapy, predict treatment outcomes, and steer therapies toward precision medicine. Future research should focus on optimizing combination regimens and determining optimal treatment cycles to achieve extended survival benefits.

In conclusion, the present meta-analysis evaluated the long-term efficacy and safety of first-line PD-1 inhibitors plus chemotherapy compared to chemotherapy alone in patients with advanced HER2-negative gastric cancer. The PD-1 inhibitors plus chemotherapy were associated with significant improvements in OS, PFS, and ORR in patients with advanced HER2-negative GC. Although the enhanced efficacy of PD-1 inhibitors plus chemotherapy was accompanied by an increased incidence of adverse events, the TRAEs observed in the PD-1 inhibitors plus chemotherapy group were manageable with appropriate interventions.

## Data Availability

The datasets presented in this study can be found in online repositories. The names of the repository/repositories and accession number(s) can be found in the article/**Supplementary Material**.
