# Supplementary material for: Long-term outcomes of PD-1 inhibitors plus chemotherapy as first-line treatment for advanced HER2-negative gastric cancer: an updated systematic review and meta-analysis
Source: Front Immunol. 2025 Nov 18;16:1651176. doi: 10.3389/fimmu.2025.1651176 (PMC12670175; doi:10.3389/fimmu.2025.1651176)
Supplement: Supplementary file 1 [file DataSheet1.pdf]

PubMed		
No.	Query	Results
#1	((((((((((((((((((((((((((Gastric Cancer[Title/Abstract]) OR (Neoplasm, Stomach[Title/Abstract])) OR (Stomach Neoplasm[Title/Abstract])) OR (Neoplasms, Stomach[Title/Abstract])) OR (Gastric Neoplasms[Title/Abstract])) OR (Gastric Neoplasm[Title/Abstract])) OR (Neoplasm, Gastric[Title/Abstract])) OR (Neoplasms, Gastric[Title/Abstract])) OR (Cancer of Stomach[Title/Abstract])) OR (Stomach Cancers[Title/Abstract])) OR (Stomach Neoplasms[Title/Abstract])) OR (Cancer, Gastric[Title/Abstract])) OR (Cancers, Gastric[Title/Abstract])) OR (Gastric Cancers[Title/Abstract])) OR (Stomach Cancer[Title/Abstract])) OR (Cancer, Stomach[Title/Abstract])) OR (Cancers, Stomach[Title/Abstract])) OR (Cancer of the Stomach[Title/Abstract])) OR (Gastric Cancer, Familial Diffuse[Title/Abstract])) OR (Carcinoma of Stomach[Title/Abstract])) OR (Stomach Carcinomas[Title/Abstract])) OR (Gastric Carcinoma[Title/Abstract])) OR (Carcinoma, Gastric[Title/Abstract])) OR (Carcinomas, Gastric[Title/Abstract])) OR (Gastric Carcinomas[Title/Abstract])) OR (Stomach Carcinoma[Title/Abstract])) OR (Carcinoma, Stomach[Title/Abstract])) OR (Carcinomas, Stomach[Title/Abstract])) OR (Carcinoma of the Stomach[Title/Abstract])) OR (Gastric Carcinoma, Familial Diffuse[Title/Abstract])	138,229
#2	((PD-1Inhibitors[Title/Abstract]) OR (PD-1 Inhibitors[Title/Abstract])) OR (PD 1 Inhibitors[Title/Abstract])) OR (PD-1 Inhibitor[Title/Abstract])) OR (Inhibitor, PD-1[Title/Abstract])) OR (PD 1 Inhibitor[Title/Abstract])) OR (Programmed Cell Death Protein 1 Inhibitor[Title/Abstract])) OR (Programmed Cell Death Protein 1 Inhibitors[Title/Abstract])) OR (Nivolumab[Title/Abstract])) OR (Opdivo[Title/Abstract])) OR (ONO-4538[Title/Abstract])) OR (ONO 4538[Title/Abstract])) OR (ONO4538[Title/Abstract])) OR (MDX-1106[Title/Abstract])) OR (MDX 1106[Title/Abstract])) OR (MDX1106[Title/Abstract])) OR (BMS-936558[Title/Abstract])) OR (BMS 936558[Title/Abstract])) OR (BMS936558[Title/Abstract])) OR (Pembrolizumab[Title/Abstract])) OR (SCH-900475[Title/Abstract])) OR (lambrolizumab[Title/Abstract])) OR (MK-3475[Title/Abstract] OR Keytruda[Title/Abstract])) OR (Tislelizumab[Title/Abstract])) OR (BGB-A317[Title/Abstract])) OR (Toripalimab[Title/Abstract])) OR (Camrelizumab[Title/Abstract])) OR (carrelizumab[Title/Abstract])) OR (SHR-1210[Title/Abstract])) OR (SHR 1210[Title/Abstract])) OR (Sintilimab[Title/Abstract])) OR (IBI 308[Title/Abstract])) OR (IBI308[Title/Abstract])) OR (IBI-308[Title/Abstract])) OR (Zimberelimab[Title/Abstract])) OR (GLS-010[Title/Abstract])) OR (Prolgolimab[Title/Abstract])) OR (Dostarlimab[Title/Abstract])) OR (Jemperli[Title/Abstract])) OR (dostarlimab-gxly[Title/Abstract])) OR (TSR-042[Title/Abstract])) OR (GSK4057190[Title/Abstract])) OR (PD-1 Blockade[Title/Abstract])) OR (Blockade, PD-1[Title/Abstract])) OR (PD 1	27,220

	Blockade[Title/Abstract])) OR (immunoglobulin G1, anti-(human CD antigen CD274) (human monoclonal MDPL3280a heavy chain), disulfide with human monoclonal MDPL3280a kappa-chain, dimer[Title/Abstract])	
#3	(((randomized controlled trial [pt] OR controlled clinical trial [pt] OR randomized [tiab] OR placebo [tiab] OR clinical trials as topic [mesh: noexp] OR randomly [tiab] OR trial [ti])	1,823,318
#4	(Advanced[Title/Abstract]) OR (Metastatic[Title/Abstract])	950,894
#5	#1 AND #2 AND #3 AND #4	196

Web of science		
No.	Query	Results
#1	TS=(Gastric Cancer OR Neoplasm, Stomach OR Stomach Neoplasm OR Neoplasms, Stomach OR Gastric Neoplasms OR Gastric Neoplasm OR Neoplasm, Gastric OR Neoplasms, Gastric OR Cancer of Stomach OR Stomach Cancers OR Stomach Neoplasms OR Cancer, Gastric OR Cancers, Gastric OR Gastric Cancers OR Stomach Cancer OR Cancer, Stomach OR Cancers, Stomach OR Cancer of the Stomach OR Gastric Cancer, Familial Diffuse OR Carcinoma of Stomach OR Stomach Carcinomas OR Gastric Carcinoma OR Carcinoma, Gastric OR Carcinomas, Gastric OR Gastric Carcinomas OR Stomach Carcinoma OR Carcinoma, Stomach OR Carcinomas, Stomach OR Carcinoma of the Stomach OR Gastric Carcinoma, Familial Diffuse)	**313,860**
#2	((TS=(PD-1Inhibitors)) OR TS=(PD-1 Inhibitors)) OR TS=(PD 1 Inhibitors)) OR TS=(PD-1 Inhibitor)) OR TS=(Inhibitor, PD-1)) OR TS=(PD 1 Inhibitor)) OR TS=(Programmed Cell Death Protein 1 Inhibitor)) OR TS=(Programmed Cell Death Protein 1 Inhibitors)) OR TS=(Nivolumab)) OR TS=(Opdivo)) OR TS=(ONO-4538)) OR TS=(ONO 4538)) OR TS=(ONO4538)) OR TS=(MDX-1106)) OR TS=(MDX 1106)) OR TS=(MDX1106)) OR TS=(BMS-936558)) OR TS=(BMS 936558)) OR TS=(BMS936558)) OR TS=(Pembrolizumab)) OR TS=(SCH-900475)) OR TS=(lambrolizumab)) OR TS=(MK-3475 OR Keytruda)) OR TS=(Tislelizumab)) OR TS=(BGB-A317)) OR TS=(Toripalimab)) OR TS=(Camrelizumab)) OR TS=(carrelizumab)) OR TS=(SHR-1210)) OR TS=(SHR 1210)) OR TS=(Sintilimab)) OR TS=(IBI 308)) OR TS=(IBI308)) OR TS=(IBI-308)) OR TS=(Zimberelimab)) OR TS=(GLS-010)) OR TS=(Prolgolimab)) OR TS=(Dostarlimab)) OR TS=(Jemperli)) OR TS=(dostarlimab-gxly)) OR TS=(TSR-042)) OR TS=(GSK4057190)) OR TS=(PD-1 Blockade)) OR TS=(Blockade, PD-1)) OR TS=(PD 1 Blockade)) OR TS=(immunoglobulin G1, anti-(human CD antigen CD274) (human monoclonal MDPL3280a heavy chain), disulfide with human monoclonal MDPL3280a kappa-chain, dimer)	**120,887**
#3	((((((((TS=(randomized controlled trial)) OR TS=(controlled clinical trial)) OR TS=(Randomized)) OR TS=(placebo)) OR TS=(clinical trials as topic)) OR TS=(randomly)) OR TS=(Trial)))	**4,329,731**
#4	TS=(Advanced OR Metastatic)	**3,604,988**
#5	#1 AND #2 AND #3 AND #4	**1,003**

Embase		
No.	Query	Results
#1	‘Gastric Cancer’:ab,ti,kw OR ‘Neoplasm, Stomach’:ab,ti,kw OR ‘Stomach Neoplasm’:ab,ti,kw OR ‘Neoplasms, Stomach’:ab,ti,kw OR ‘Gastric Neoplasms’:ab,ti,kw OR ‘Gastric Neoplasm’:ab,ti,kw OR ‘Neoplasm, Gastric’:ab,ti,kw OR ‘Neoplasms, Gastric’:ab,ti,kw OR ‘Cancer of Stomach’:ab,ti,kw OR ‘Stomach Cancers’:ab,ti,kw OR ‘Stomach Neoplasms’:ab,ti,kw OR ‘Cancer, Gastric’:ab,ti,kw OR ‘Cancers, Gastric’:ab,ti,kw OR ‘Gastric Cancers’:ab,ti,kw OR ‘Stomach Cancer’:ab,ti,kw OR ‘Cancer, Stomach’:ab,ti,kw OR ‘Cancers, Stomach’:ab,ti,kw OR ‘Cancer of the Stomach’:ab,ti,kw OR ‘Gastric Cancer, Familial Diffuse’:ab,ti,kw OR ‘Carcinoma of Stomach’:ab,ti,kw OR ‘Stomach Carcinomas’:ab,ti,kw OR ‘Gastric Carcinoma’:ab,ti,kw OR ‘Carcinoma, Gastric’:ab,ti,kw OR ‘Carcinomas, Gastric’:ab,ti,kw OR ‘Gastric Carcinomas’:ab,ti,kw OR ‘Stomach Carcinoma’:ab,ti,kw OR ‘Carcinoma, Stomach’:ab,ti,kw OR ‘Carcinomas, Stomach’:ab,ti,kw OR ‘Carcinoma of the Stomach’:ab,ti,kw OR ‘Gastric Carcinoma, Familial Diffuse’:ab,ti,kw	**160,145**
#2	'pd-1 inhibitors':ab,ti,kw OR 'pd 1 inhibitors':ab,ti,kw OR 'pd-1 inhibitor':ab,ti,kw OR 'inhibitor, pd-1':ab,ti,kw OR 'pd 1 inhibitor':ab,ti,kw OR 'programmed cell death protein 1 inhibitors':ab,ti,kw OR 'programmed cell death protein 1 inhibitor':ab,ti,kw OR pembrolizumab:ab,ti,kw OR 'sch 900475':ab,ti,kw OR lambrolizumab:ab,ti,kw OR 'mk 3475':ab,ti,kw OR keytruda:ab,ti,kw OR nivolumab:ab,ti,kw OR opdivo:ab,ti,kw OR 'ono 4538':ab,ti,kw OR 'mdx 1106':ab,ti,kw OR mdx1106:ab,ti,kw OR 'bms 936558':ab,ti,kw OR bms936558:ab,ti,kw OR toripalimab:ab,ti,kw OR 'bgb a317':ab,ti,kw OR camrelizumab:ab,ti,kw OR carrelizumab:ab,ti,kw OR 'shr 1210':ab,ti,kw OR 'gls 010':ab,ti,kw OR cemiplimab:ab,ti,kw OR regn2810:ab,ti,kw OR sintilimab:ab,ti,kw OR ibi308:ab,ti,kw OR 'ibi 308':ab,ti,kw OR zimberelimab:ab,ti,kw OR prolgolimab:ab,ti,kw OR dostarlimab:ab,ti,kw OR jemperli:ab,ti,kw OR 'dostarlimab gxly':ab,ti,kw OR 'tsr 042':ab,ti,kw OR ‘gsk4057190’:ab,ti,kw OR ‘PD-1 Blockade’:ab,ti,kw OR ‘Blockade, PD-1’:ab,ti,kw OR ‘PD 1 Blockade’:ab,ti,kw OR ‘immunoglobulin G1, anti-(human CD antigen CD274) (human monoclonal MDPL3280a heavy chain), disulfide with human monoclonal MDPL3280a kappa-chain, dimer’:ab,ti,kw	**63,857**
#3	'randomized controlled trial':ab,ti,kw OR 'controlled clinical trial':ab,ti,kw OR randomized:ab,ti,kw OR placebo:ab,ti,kw OR 'clinical trials as topic':ab,ti,kw OR randomly:ab,ti,kw OR trial:ab,ti,kw	**2,778,813**
#4	‘Advanced’:ab,ti,kw OR ‘Metastatic’:ab,ti,kw	**1,477,449**
#5	#1 AND #2 AND #3 AND #4	**771**

Cochrane library

NO.	Query	Results
#1	(Gastric Cancer OR Neoplasm, Stomach OR Stomach Neoplasm OR Neoplasms, Stomach OR Gastric Neoplasms OR Gastric Neoplasm OR Neoplasm, Gastric OR Neoplasms, Gastric OR Cancer of Stomach OR Stomach Cancers OR Stomach Neoplasms OR Cancer, Gastric OR Cancers, Gastric OR Gastric Cancers OR Stomach Cancer OR Cancer, Stomach OR Cancers, Stomach OR Cancer of the Stomach OR Gastric Cancer, Familial Diffuse OR Carcinoma of Stomach OR Stomach Carcinomas OR Gastric Carcinoma OR Carcinoma, Gastric OR Carcinomas, Gastric OR Gastric Carcinomas OR Stomach Carcinoma OR Carcinoma, Stomach OR Carcinomas, Stomach OR Carcinoma of the Stomach OR Gastric Carcinoma, Familial Diffuse):ti,ab,kw	**13,377**
#2	(PD-1Inhibitors OR PD-1 Inhibitors OR PD 1 Inhibitors OR PD-1 Inhibitor OR Inhibitor, PD-1 OR PD 1 Inhibitor OR Programmed Cell Death Protein 1 Inhibitor OR Programmed Cell Death Protein 1 Inhibitors OR Nivolumab OR Opdivo OR ONO-4538 OR ONO 4538 OR ONO4538 OR MDX-1106 OR MDX 1106 OR MDX1106 OR BMS-936558 OR BMS 936558 OR BMS936558 OR Pembrolizumab OR SCH-900475 OR lambrolizumab OR MK-3475 OR Keytruda OR Tislelizumab OR BGB-A317 OR Toripalimab OR Camrelizumab OR carrelizumab OR SHR-1210 OR SHR 1210 OR Sintilimab OR IBI 308 OR IBI308 OR IBI-308 OR Zimberelimab OR GLS-010 OR Prolgolimab OR Dostarlimab OR Jemperli OR dostarlimab-gxly OR TSR-042 OR GSK4057190 OR PD-1 Blockade OR Blockade, PD-1 OR PD 1 Blockade OR immunoglobulin G1, anti-(human CD antigen CD274) (human monoclonal MDPL3280a heavy chain), disulfide with human monoclonal MDPL3280a kappa-chain, dimer):ti,ab,kw	**15,062**
#3	(randomized controlled trial OR controlled clinical trial OR Randomized OR placebo OR clinical trials as topic OR randomly OR Trial):ti,ab,kw	**1,631,606**
#4	(Advanced OR Metastatic):ti,ab,kw	**101,166**
#5	#1 AND #2 AND #3 AND #4	**487**
