# Supplementary material for: Long-term outcomes of PD-1 inhibitors plus chemotherapy as first-line treatment for advanced HER2-negative gastric cancer: an updated systematic review and meta-analysis
Source: Front Immunol. 2025 Nov 18;16:1651176. doi: 10.3389/fimmu.2025.1651176 (PMC12670175; doi:10.3389/fimmu.2025.1651176)

Supplement Figure Legends

Figure S1. funnel plot for OS

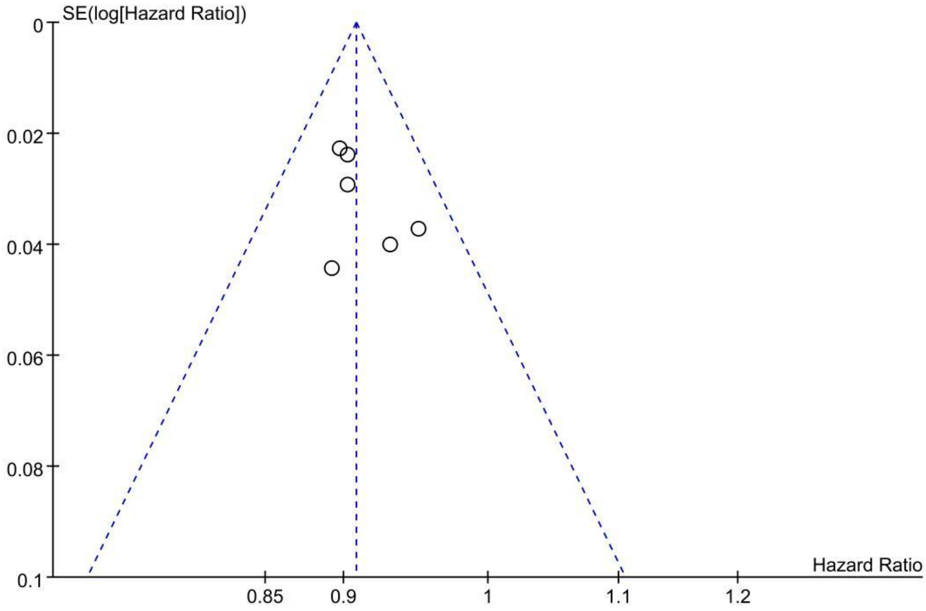

Figure S2. funnel plot for PFS

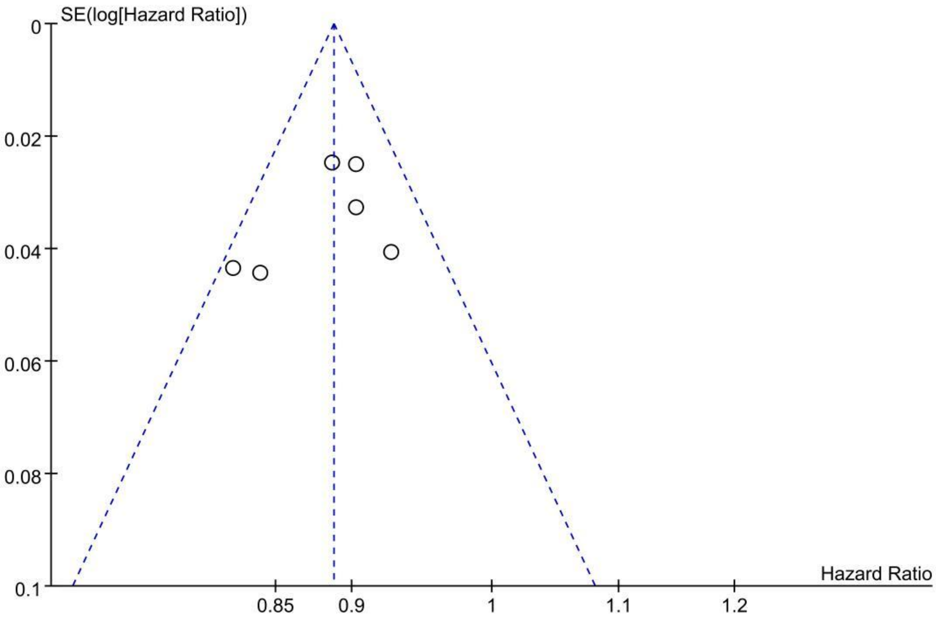

Figure S3. funnel plot for ORR

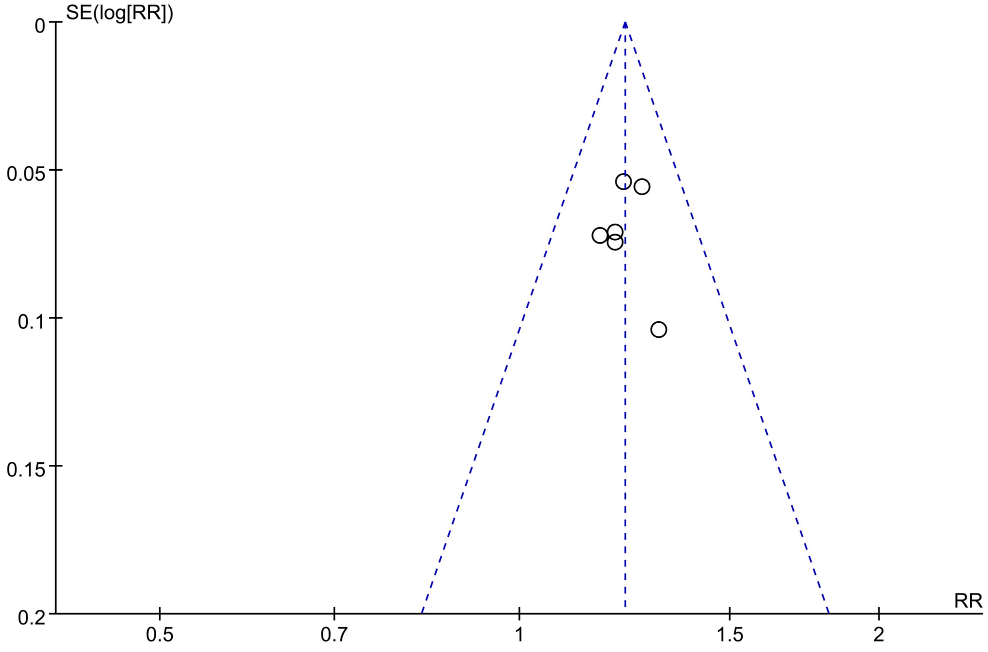

Figure S4. funnel plot for TRAEs

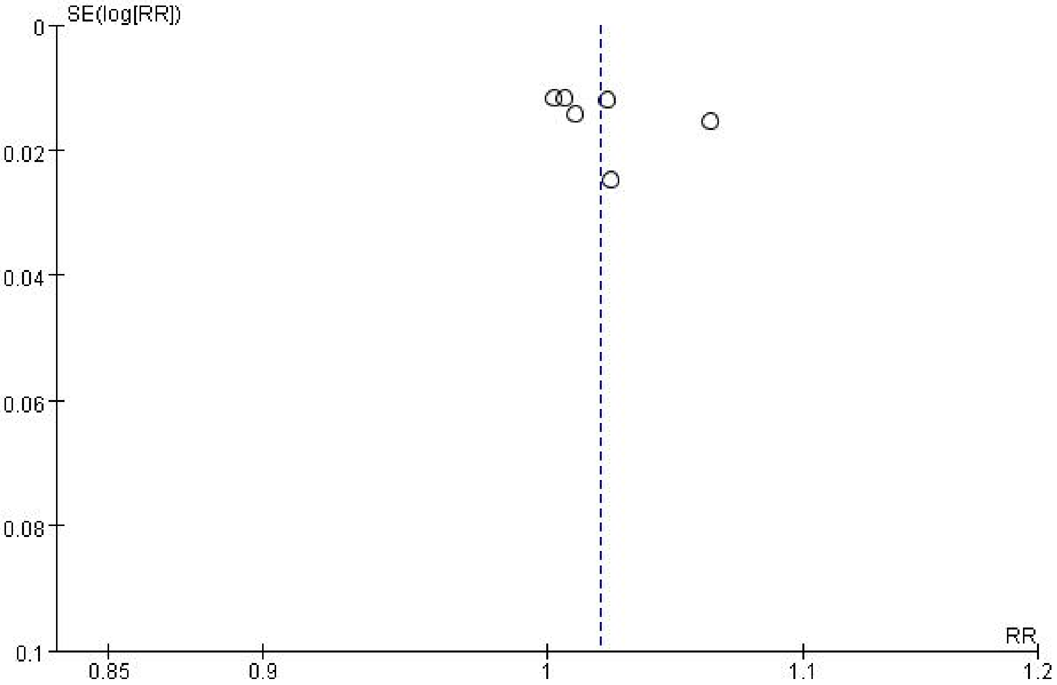

Figure S5. funnel plot for Grade≥3 TRAEs

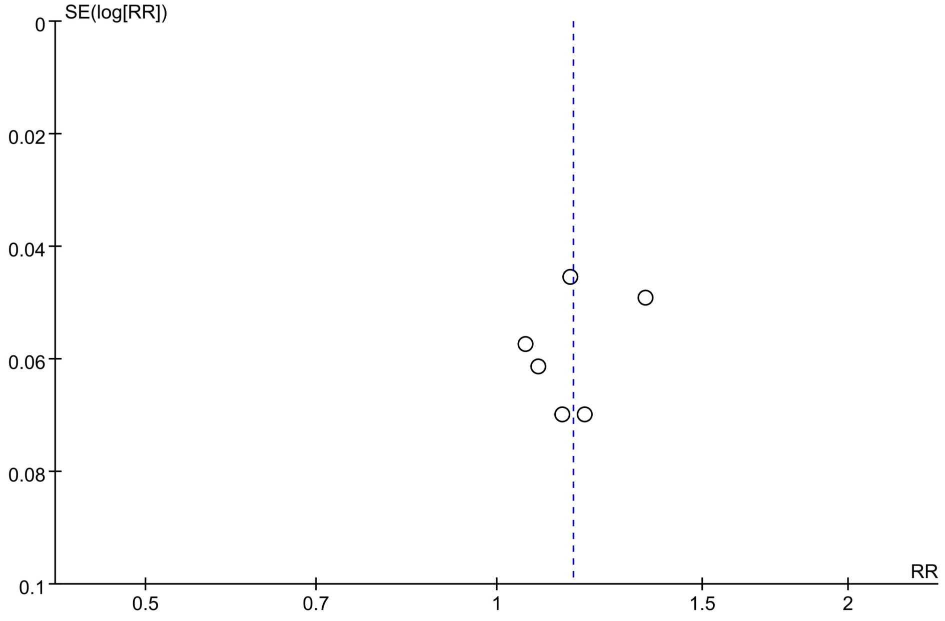

Supplement: Supplementary file 2 [file DataSheet2.pdf]
